# Genome-assisted prediction of a quantitative trait measured in parents and progeny: application to food conversion rate in chickens

**DOI:** 10.1186/1297-9686-41-3

**Published:** 2009-01-05

**Authors:** Oscar González-Recio, Daniel Gianola, Guilherme JM Rosa, Kent A Weigel, Andreas Kranis

**Affiliations:** 1 Department of Dairy Science, University of Wisconsin, Madison, WI 53706, USA; 2 Department of Animal Sciences, University of Wisconsin, Madison, WI 53706, USA; 3 Aviagen Ltd., Newbridge, Scotland, UK

## Abstract

Accuracy of prediction of yet-to-be observed phenotypes for food conversion rate (FCR) in broilers was studied in a genome-assisted selection context. Data consisted of FCR measured on the progeny of 394 sires with SNP information. A Bayesian regression model (Bayes A) and a semi-parametric approach (Reproducing kernel Hilbert Spaces regression, RKHS) using all available SNPs (*p *= 3481) were compared with a standard linear model in which future performance was predicted using pedigree indexes in the absence of genomic data. The RKHS regression was also tested on several sets of pre-selected SNPs (*p *= 400) using alternative measures of the information gain provided by the SNPs. All analyses were performed using 333 genotyped sires as training set, and predictions were made on 61 birds as testing set, which were sons of sires in the training set. Accuracy of prediction was measured as the Spearman correlation (${\bar{r}}_{S}$) between observed and predicted phenotype, with its confidence interval assessed through a bootstrap approach. A large improvement of genome-assisted prediction (up to an almost 4-fold increase in accuracy) was found relative to pedigree index. Bayes A and RKHS regression were equally accurate (${\bar{r}}_{S}$ = 0.27) when all 3481 SNPs were included in the model. However, RKHS with 400 pre-selected informative SNPs was more accurate than Bayes A with all SNPs.

## Introduction

Genome-wide association studies of diseases and complex traits [1] have permeated into animal breeding, and genome-assisted selection has become a major focus of research [2,3]. However, genome-based artificial selection poses several challenges. For instance, methods for prediction of genetic merit or phenotype using a large number of markers must be contrasted and improved. Also, biological and economical advantages of genome-assisted selection in a breeding program must be quantified (this second problem is not addressed herein).

A very important issue is how to deal with a much larger number of markers (*p*) than of individuals that are genotyped (*n*). Some proposals include treating marker effects as random, with shrinkage of estimates of non-informative markers to zero. This is done naturally in a Bayesian context, where all unknowns are treated as random variables (*e.g*., Gianola and Fernando, [4]). On the one hand, Bayesian regression methods, such as Bayes A and Bayes B [2], or the special case of Bayes A described by Xu [5] have recently gained attention. However, all of these procedures involve strong assumptions a priori. On the other hand, non-parametric methods have been suggested as an alternative for predicting genomic breeding values, because these methods may require weaker assumptions when modeling complex quantitative traits [6].

These non-parametric approaches have been applied to simulated [7] and field [8] data, and results seem promising. The simulations from Gianola *et al*. [7] involved 100 biallelic markers and additive × additive interactions between five pairs of loci. Gonzalez-Recio *et al*. [8] used 24 pre-selected SNPs from a filter and wrapper feature subset selection algorithm [9] in a reproducing kernel Hilbert spaces (RKHS) regression model. However, these non-parametric methods have not been tested yet using a large number of SNPs and field data. Inclusion of a large number of SNPs in these non-parametric models must be studied. Further, evaluating the accuracy of such methods in predicting phenotypes in future generations is a crucial issue in artificial selection programs.

Genomic information became available in animal breeding recently, and most research involving either the large *p *small *n *problem [10,2] or the prediction of future generations [11,12] has resorted to simulations. Arguably, assumptions built in simulations may fail to represent the true complexity of biological systems and, typically, simulations tend to favor some of the models under evaluation. Therefore, the extent to which simulation results hold with real data can be questioned.

The present paper uses field data from the Genomics Initiative Project at Aviagen Ltd. (Newbridge, UK). Food conversion rate (**FCR**) is one of the most economically important traits in the broiler industry, because it affects feeding and housing costs markedly. Genome-assisted selection programs may provide greater reliability of predictions of future performance, thus increasing profitability.

The objective of this study was to compare the ability of Bayes A regression and of semi-parametric (RKHS) regression to predict yet-to-be observed phenotypes, using field data on FCR in a two-generation setting.

## Methods

Animal Care and Use Committee approval was not obtained for this study because the data were obtained from an existing database supplied by Aviagen Ltd. (Newbridge, UK).

In a nutshell, a one-fold cross-validation with a training set and a testing set was carried out, as the testing set included only sons of sires that were in the training test. Several statistical methods were used to predict the average phenotypes of offspring of animals in the testing set, *i.e*., first-generation performance. These included a standard genetic evaluation, which ignored SNP genotypes, and two methods that included all available SNPs (after editing) as predictors in the model. The latter methods, which included genomic information, were Bayesian regression and RKHS regression. In addition, the RKHS regression approach was fitted with 400 pre-selected SNPs, where pre-selection was based on information gain using alternative criteria. In this section, the data set employed, the pre-selection of SNPs, and the statistical methods that were applied are described.

### Phenotypic Data

Data consisted of average FCR records for progeny of each of 394 sires from a commercial broiler line in the breeding program of Aviagen Ltd. Prior to the analyses, the individual bird FCR records were adjusted for environmental and mate effects, as described in Ye *et al*. [13]. In order to assess the reliability of genome-assisted evaluation, two data sets (training and testing) were constructed. The testing set included offspring from sires with records in the training set. Sires included in the testing set were required to have sires in the training set with progeny records, and needed to have more than 20 progenies with FCR records, to have a reliable mean phenotype. Sires in the training and testing sets had an average of 33 and 44 progeny, respectively. Family size (half sibs) in the training set ranged between 1 and 284, with the mean and median being 32 and 17, respectively. Sixty-one sires (15.5% of the total) were included in the testing set, whereas the remaining 333 sires were in the training set. Predictions were calculated from the training set, and the accuracy of predicting the mean progeny phenotype was assessed using sons in the testing set.

### Genomic Data

Genotypes consisted of 4505 SNPs chosen from the 2.8 million SNPs identified in the sequencing project of the chicken genome [14]. A data file titled "Database of SNPs used in the Illumina Corp. chicken genotyping project" (downloadable from http://poultry.mph.msu.edu/resources/resources.htm) describes partially the panel used, and further details on the 6 K panel can be found in Andreescu *et al*. [15]. All SNPs with monomorphic genotypes or with minor allele frequencies less than 5% were excluded. After editing, genotypes consisted of 3481 of the initial 4505 SNPs.

Pre-selection of SNPs to be included in the analyses was performed using the information gain or entropy reduction criterion [16,9]. Information gain is the difference in entropy of a probability distribution before and after observing genotypes, *i.e*., it measures how much uncertainty is reduced by observation of SNP genotypes. The entropy of the probability distribution of a discrete random variable Y is defined as:

$$H(\mathrm{Pr}(Y))=-\sum_{y\in A}\mathrm{Pr}(y)\log_{2}\mathrm{Pr}(y),$$

where A is the set of all states that Y can take, and the logarithm is on base 2 to mimic bits of information. The above pertains to a discrete distribution since entropy is not well defined in the continuous case [17]. Here, Y refers to FCR phenotypes that were discretized by considering different number of classes of FCR and different cutpoints, as follows. First, two extreme FCR classes ("low" and "high") were set up using cutpoints corresponding to the α and (1-α) quantiles (α = 0.15, 0.20, 0.25, 0.35 and 0.40) of the FCR phenotypes for sires in the training set. Further, an additional "middle" class (FCR between percentiles 0.40 and 0.60) was included to enrich the discretized data. In total, information gain was calculated in ten subsets, corresponding to combinations of the five 'extreme' tail α-values, with or without the intermediate class.

For each SNP, the training set was divided into three subsets corresponding to the three possible genotypes (aa, Aa or AA). For each genotype *k *there are $N_{k}^{H}$ sires with genotype *k *in the high class, $N_{k}^{L}$ sires with genotype *k *in the low class, and possibly $N_{k}^{M}$ sires with genotype *k *in the middle class, if included. The information gain for each SNP *s *(*s *= 1,2, ..., 3481) was the change in entropy after observing the genotypes, calculated as:

$$IG(SNP_{i})=H(\mathrm{Pr}(Y))-\sum_{k=1}^{3}\left(\frac{\sum_{C=L,M,H}N_{k}^{C}}{N}\left(-\sum_{C=L,M,H}\frac{N_{k}^{C}}{N}\log_{2}\frac{N_{k}^{C}}{N}\right)\right),$$

where $\text{N}=N_{k}^{L}+N_{k}^{M}+N_{k}^{H}$. Note that $N_{k}^{M}$ = 0 if a middle class was not included.

The 400 SNPs with largest information gain in each of the ten partitions were pre-selected to build up a 400-SNP genotype for each sire. Note that the choice of the 400 SNPs was arbitrary, but it roughly represents 10% of the initial SNPs.

### Models

Let **y **(333 × 1) be the vector of mean adjusted FCR records for progeny of sires in the training set. Three different methods for prediction of genomic breeding values for FCR were used, as described next.

#### Standard genetic evaluation (E-BLUP)

A Bayesian equivalent of empirical best linear unbiased prediction of sires' transmitting abilities, as described by Henderson [18], was used. This method uses pedigree data as the only source of genetic information. The linear model was:

**y **= *μ***1 **+ **Zu **+ **e**

where, *μ *is an unknown mean; **1 **is a vector of ones; **u **= {*u*_*i*_} is a vector of sire effects; *u*_*i *_is the effect of sire *i *in the pedigree (*i *= 1, 2, ..., 624) and **Z **is an incidence matrix of order 333 × 624 linking **u **to the observed data. A priori, the sire effects were assumed to be distributed as **u **~*N*(**0**, **A**$\sigma_{u}^{2}$), where **A **is the additive relationship matrix between sires, and $\sigma_{u}^{2}$ is the variance between sires. The residuals, **e**, were assumed to be distributed as *N*(**0**, **R **= **N**^-1 ^$\sigma_{e}^{2}$), where **N **= {*n*_*i*_} is a diagonal matrix with elements *n*_*i *_representing the number of progeny of sire *i *and $\sigma_{e}^{2}$ is the residual variance. This dispersion structure for **e **weights the residuals according to the number of progeny each sire has [17,19]. Independent scaled inverted chi-square prior distributions were assigned to the sire and residual variances as $\sigma_{u}^{2}\sim \upsilon_{u}s_{u}^{2}\chi_{\upsilon_{u}}^{-1}$ and $\sigma_{e}^{2}\sim \upsilon_{e}s_{e}^{2}\chi_{\upsilon_{e}}^{-1}$, respectively, where *υ*_*u *_= 5 and *υ*_*e *_= 3 correspond to the degrees of freedom, and $s_{u}^{2}$ = 0.1 and $s_{e}^{2}$ = 8.67 were the corresponding scale parameters. Sire merit (transmitting ability) was inferred using a Gibbs sampling algorithm.

#### Bayes A

Meuwissen *et al*. [2] have proposed a Bayesian model in which the additive effects of chromosome segments marked by SNPs follow a normal distribution with a segment-specific variance. These variances are assigned a common scaled inverted chi-square prior distribution. The model fitted in this study had the form:

**y **= *μ***1 **+ **Xb **+ **e**.

Here, **y **is a 333 × 1 vector of progeny means for adjusted FCR, *μ *is their mean value, and **1 **is a column vector of ones; **b **= {*b*_*s*_} is a vector of 3481 × 1 SNP effects, and *b*_*s *_is the regression coefficient on the additive effect of SNP *s *(*s *= 1, 2, ..., 3481). Elements of the incidence matrix **X**, of order *n *× *p *(*n *= 333; *p *= 3481), were set up as for an additive model, with values -1, 0 or 1 for *aa*, *Aa *and *AA*, respectively. The *b*_*s *_effects were assumed normally and independently distributed a priori as N(0, $\sigma_{s}^{2}$), where $\sigma_{s}^{2}$ is an unknown variance specific to marker *s*. The prior distribution of each $\sigma_{s}^{2}$ was assumed to be *χ*^-2^(*υ*, *S*) with *υ *= 4 and *S *= 0.01. The residuals (**e**) were assumed to be distributed as *N*(**0**, **R**), with **R **constructed as in the previous model.

#### Reproducing kernel Hilbert spaces regressions

A RKHS regression [20-22] is a semi-parametric approach that allows inference regarding functions, e.g., genomic breeding values, without making strong prior assumptions. As described in Gianola and van Kaam [6] and González-Recio *et al*. [8] in the context of genome-assisted selection, this model can be formulated as:

**y **= **Xβ **+ **K**_*h*_**α **+ **e**,

where the first term (**Xβ**) is a parametric term with β as a vector of systematic effects or nuisance parameters (only *μ *was fitted in this case, since the data were pre-corrected), and **X **is an incidence matrix (here a vector of ones, **1**). The non-parametric term is given by **K**_*h*_**α**, where **K**_*h *_is a positive definite matrix of kernels, possibly dependent on a bandwidth parameter (*h*), and α is vector of non-parametric coefficients that are assumed to be distributed as $\alpha \sim N(0,K_{h}^{-1}\sigma_{\alpha }^{2})$, with $\sigma_{\alpha }^{2}$ representing the reciprocal of a smoothing parameter ($\sigma_{\alpha }^{2}$ = *λ*^-1^). The residuals **e **were assumed to be distributed as **e **~*N*(0, **R**), with **R **as for the previous models. It can be shown that, given *h *and *λ*, the RKHS regression solutions satisfy the linear system:

$$\left[\begin{array}{cc} 1^{\prime }R^{-1}1 & 1^{\prime }R^{-1}K_{h}\\ {K^{\prime }}_{h}R^{-1}1 & {K^{\prime }}_{h}R^{-1}K_{h}+\frac{1}{\lambda^{-1}}K_{h} \end{array}\right]\left[\begin{array}{c} {\hat{\mu }}_{\lambda }\\ {\hat{\alpha }}_{\lambda } \end{array}\right]\left[\begin{array}{c} 1^{\prime }R^{-1}y\\ {K^{\prime }}_{h}R^{-1}y \end{array}\right].$$

There are two key issues in the RKHS regression pertaining to the non-parametric term: choosing the matrix of kernels, and tuning the *h *and *λ *parameters. The matrix of kernels aims to measure "distances" between genotypes. This matrix **K**_*h *_had dimension 333 × 333, with rows in the form ${k^{\prime }}_{i}=\left\{K_{h}(x_{i}-x_{j})\right\}$, *j *= 1, 2, ..., 333, where *K*_*h*_(**x**_*i *_- **x**_*j*_) is the kernel involving the genotypes of sires *i *and *j*. The kernel refers to any smooth function for distances between objects, such that *K*_*h*_(**x**_*i *_- **x**_*j*_) ≥ 0. Different types of kernels may be used [23]. A Gaussian kernel was chosen in this research, with form: $K_{h}(x_{i}-x_{j})=\exp\left(-\frac{dist(x_{i}-x_{j})}{h}\right)$, where *dist*(**x**_*i *_- **x**_*j*_) is a measurement of distance between genotypes of sires *i *and *j*, and *h *is a bandwidth parameter. The choice of *h *and of the measurement of distance between genotypes must be done cautiously. A generalized (direct) cross validation procedure was used to tune *h*, as described in Wahba *et al*. [24]. However, measuring distances between genotypes is less straightforward, because a large variety of criteria might be used for this purpose (e.g. Gianola *et al*. [7]; Gianola and van Kaam, [6]; Gonzalez-Recio *et al*., [8]).

The algorithm used to measure distances between genotypes is given next. Let **x**_*i *_and **x**_*j *_be string sequences of SNP genotypes for sires *i *and *j*, respectively. These strings can be separated into *m *substrings in which all SNPs differ between the two sequences. For example, suppose **x**_*i *_= (AABbCCDDEeFFGg), and **x**_*j *_= (AabbCcDDEeffgg). Here, there are two substrings that differ from each other completely, corresponding to SNPs from loci 1–3, and 6–7 (table 1).

**Table 1:** Two substrings that differ from each other completely, corresponding to SNPs from loci 1–3, and 6–7

	Substring 1	Substring 2
**Sire *i***	AABbCC	FFGg
**Sire *j***	AabbCc	ffgg

Then, compute the sum of the logarithms in base 2 (interpreted as bits of information) of the dissimilarity between substrings. Dissimilarity was defined as the number of alleles differing at each SNP. Hence, distance between two genotypes can be expressed as:

$$dist(x_{i}-x_{j})=\sum_{k=1}^{m}\log_{2}\left(1+DA_{k}\right),$$

where *DA*_*k *_is the number of different alleles in substring *k*. In the example, sires *i *and *j *differ in one allele at each SNP (AA *vs *Aa, Bb *vs *bb, and CC *vs *Cc) in substring 1. In substring 2, sires *i *and *j *differ in 2 alleles for the first SNP (FF *vs *ff) and in 1 allele for the second SNP (Gg *vs *gg). Here, the two substrings had distances *DA*_1 _= *DA*_2 _= 3.

A modification of this system was used for models in which SNPs were pre-selected using the information gain score. Here, the number of different alleles at each SNP was weighted by the information gain score at that locus. Therefore, the distance between two genotypes was calculated as: $dist(x_{i}-x_{j})=\sum_{k=1}^{m}\log_{2}\left(1+{w^{\prime }}_{k}da_{k}\right)$, where **w**_*k *_and **da**_*k *_are column vectors with typical elements equal to the information gain score and the number of different alleles, respectively, for each SNP in substring *k*. This kernel weights dissimilarity between SNPs by the reduction in entropy. With this approach, the kernel matrix **K **is symmetric and positive definite, so it fulfills the requirements of a RKHS, and it can be viewed as a correlation matrix between genomic combinations.

In total, 11 RKHS regression analyses were performed: one including all 3481 SNPs; five (α = 0.15, 0.20, 0.25, 0.35 and 0.40) including 400 pre-selected SNPs using the information gain calculated using two ("low" and "high") classes to classify sires, and five (α = 0.15, 0.20, 0.25, 0.35 and 0.40) including 400 pre-selected SNPs with information gain calculated by classifying sires into three ("low", "medium" and "high") classes.

Posterior estimates from all models were obtained with a Gibbs sampling algorithm based on 150,000 iterations, discarding the first 50,000 as burn-in, and keeping all 100,000 subsequent samples for inferences.

### Predictive ability

Progeny phenotypes in the testing set were predicted using the estimates obtained from the training set. First, using the training set with each model, inferences were made regarding the predicted transmitting ability for E- BLUP, prediction of SNPs coefficients for Bayes A, and prediction of non-parametric coefficients for RKHS regression. Phenotypes in the testing set were predicted as follows:

#### E-BLUP

Phenotypes were predicted using pedigree indexes via sire and maternal grandsire (information from maternal relatives was not included). The pedigree index for sire *t *in the testing set (*PI*_*t*_) was PIt=12PTAs+14PTAmgs, where *PTA*_*s *_and *PTA*_*mgs *_are the predicted transmitting abilities of the bird's sire and maternal grandsire, respectively.

#### Bayes A

The *p *= 3481 estimates of regressions coefficients corresponding to additive effects of the SNPs ($\hat{b}$) from the training set were multiplied by their respective genotype codes (*x*_*ts *_= -1, 0 or 1 for aa, Aa or AA, respectively) for sire *t *in the testing set to obtain a predicted phenotype as ${\hat{y}}_{t}=\hat{\mu }+\sum_{s=1}^{3481}{\hat{b}}_{s}x_{ts}$, where $\hat{\mu }$ and ${\hat{b}}_{s}$ are the posterior means of *μ *and *b*_*s*_, respectively.

#### Reproducing kernel Hilbert spaces regressions

Predictions were made using a matrix of kernels between focal points (i.e., genotypes of sires in the testing set) and support vectors (genotypes of sires in the training set) as:

$$\hat{y}=\hat{\mu }1+K^{*}(h)\hat{\alpha }$$

where K* (*h*) is a matrix with dimension 61 × 333, and with rows of the form ${k^{\prime }}_{t}^{*}=\left\{K_{h}^{*}(x_{t}-x_{j})\right\}$, *j *= 1, 2, ..., 333, where $K_{h}^{*}(x_{t}-x_{j})$ is the kernel between the genotype of sire *t *in the testing set and sire *j *in the training set. The same bandwidth parameter that was tuned with the training set was used. The vector $\hat{\alpha }$ represented the posterior means of the 333 non-parametric regression coefficients for sires in the training sample.

Typically, the objective of prediction in animal breeding is to rank candidates for selection, and to subsequently choose the highest-ranked candidates as parents of the next generation. Spearman correlations (r_S_) were calculated between predicted and observed phenotypes of sires in the testing set for all methods. Confidence intervals of the correlation estimates were formed using bootstrapping [25,26] for each method. Pairs, defined as the predicted phenotypes in the testing set and its corresponding observed (known) phenotype, were assumed to be from an independent and identically distributed population. Then, 10,000 pairs were drawn with replacement from the whole testing set, and the Spearman correlation was computed in each of the bootstrap samples.

Further, computing times for running the first 10,000 samples were tested for Bayes A and for RKHS regression using all 3481 SNPs in a HPxw6000 workstation with a 2.4 GHz × 2 processor and 2 Gb RAM. A Gauss-Seidel algorithm with residual updates [27] was used in the Bayes A method, as suggested by Legarra and Misztal [28]. The solving effect-by-effect strategy described in Misztal and Gianola [29] was adapted to compute the RKHS regressions.

## Results and discussion

Mean adjusted FCR was 1.23 in the training set, with a standard deviation of 0.1. The posterior mean of heritability was 0.21 with the E-BLUP model. This estimate was similar to those reported by Gaya *et al*. [30] and Pym and Nicholls [31], but higher than that of Zhang *et al*. [32]. The posterior mean (standard deviation) of the residual variance was estimated at 1.17 (0.22) and 0.50 (0.12) with Bayes A and RKHS, respectively, using all 3481 SNPs in each case. Notably, analyses using RKHS regression on 400 pre-selected SNPs produced a slightly smaller posterior mean of the residual variance than analyses based on all 3481 SNPs.

**Figure 1:**
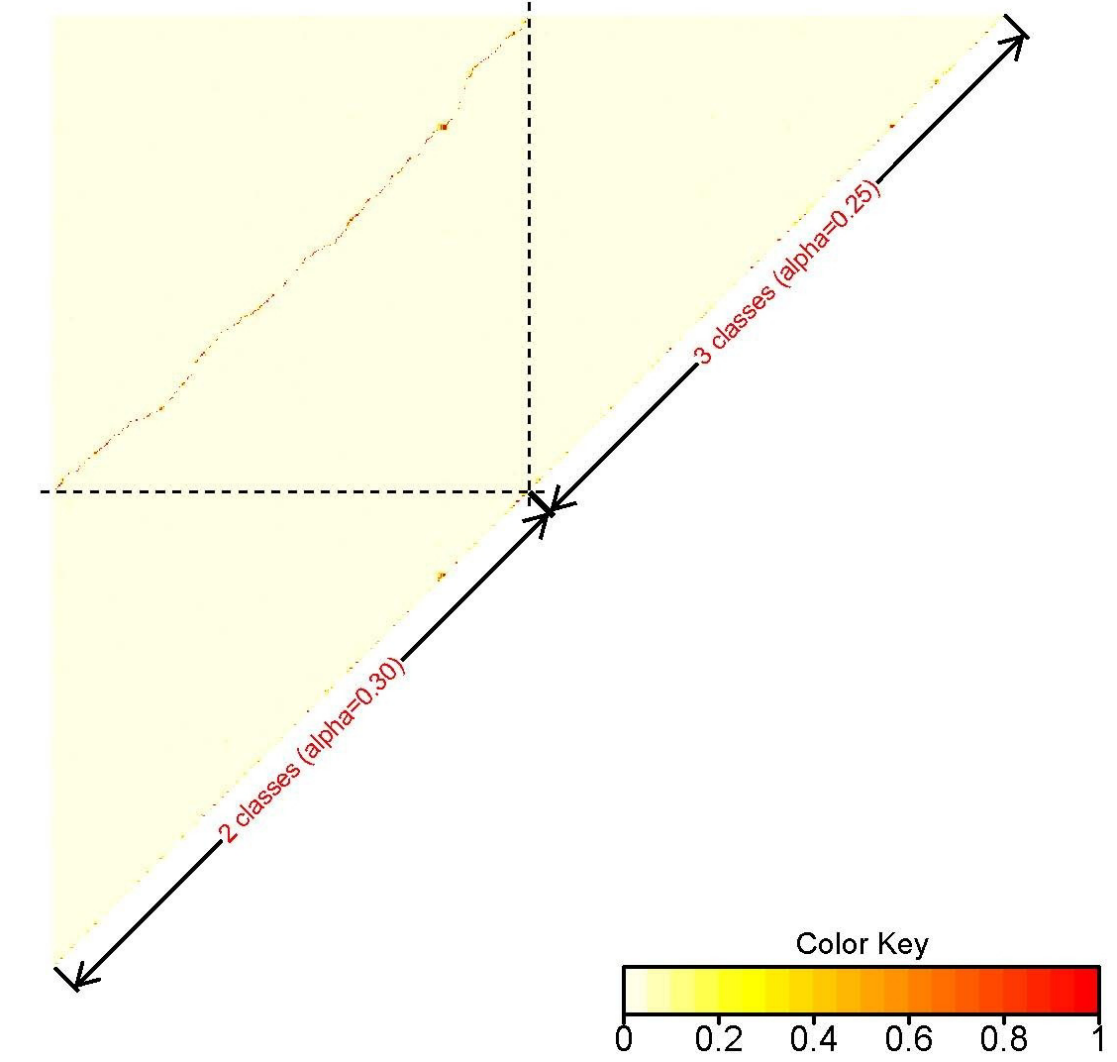
**Heat map of linkage disequilibrium (r^2^) within and between SNPs pre-selected using two different criteria for classifying sires: two classes (high and low) with quantile = 0.30 and three classes (high, medium and low) with quantile = 0.25**.

Almost half of the 400 pre-selected SNPs were selected consistently, regardless of the criterion used for classifying sires. About 60% of the remaining SNPs were in strong linkage disequilibrium (LD), measured with the r^2 ^statistic, between criterions. The most discrepant case (2 classes and α = 0.30, vs. 3 classes and α = 0.25) is shown in Figure 1 (LD between and within selected SNPs from each criterion). This figure contains the 400 SNPs selected with each of those cases. For each case, the SNPs are sorted according their position in the genome. This map shows that most of the SNPs that were pre-selected with one criterion had strong "proxy" SNPs that were pre-selected with the other criterion, as the dark points in the diagonal of the left-upper square indicate. Physical locations in the genome were also close (results not shown).

**Figure 2:**
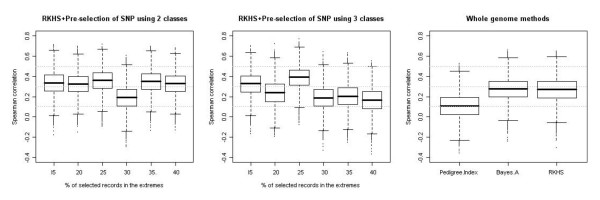
**Box plots for the bootstrap distribution of Spearman correlations between predicted and observed phenotype in the testing set (progeny) obtained with: RKHS on 400 pre-selected SNPs using two or three classes to classify sires with different percentiles (left and middle panels, respectively) and methods using pedigree or all available SNPs (right panel)**.

Table 2 and Figure 2 show descriptive statistics (mean, standard deviation, and confidence interval) and box plots, respectively, of the bootstrap distribution of Spearman correlations. The pedigree index (E-BLUP) was the least accurate predictor of phenotypes in the testing set (${\bar{r}}_{S}$ = 0.11). All analyses using genomic information outperformed E-BLUP. Results for Bayes A and RKHS regression using all available SNPs were similar, attaining an average Spearman correlation of 0.27. Size of confidence regions was similar as well.

**Table 2:** Means, standard deviations (s.d.) and 95% confidence intervals (CI) of the Bootstrap distribution of Spearman correlations between predicted and observed phenotypes in the testing set

Whole genome methods			
**Method**	**Mean**	**s.d**.	**CI (95%)**

E-BLUP	0.11	0.13	(-0.13, 0.35)
Bayes A	0.27	0.12	(0.04, 0.49)
RKHS	0.27	0.12	(0.03, 0.50)

**Information gain using 2 classes (400 pre-selected SNPs) + RKHS**			

**Quantile**	**Mean**	**s.d**.	**CI (95%)**

0.15	0.33	0.12	(0.09, 0.56)
0.20	0.32	0.11	(0.10, 0.53)
0.25	0.36	0.11	(0.13, 0.57)
0.30	0.19	0.12	(-0.05, 0.42)
0.35	0.35	0.11	(0.12,0.55)
0.40	0.33	0.11	(0.10, 0.53)

**Information gain using 3 classes (400 pre-selected SNPs) + RKHS**			

**Quantile**	**Mean**	**s.d**.	**CI (95%)**

0.15	0.32	0.11	(0.10, 0.54)
0.20	0.24	0.13	(-0.01, 0.48)
0.25	0.39	0.11	(0.16, 0.59)
0.30	0.19	0.12	(-0.05, 0.42)
0.35	0.20	0.12	(-0.04, 0.43)
0.40	0.16	0.12	(-0.08, 0.40)

E-BLUP: Bayesian linear model; Bayes A: Bayesian regression on SNP; RKHS: reproducing kernel Hilbert spaces regression

RKHS regression with pre-selected SNPs was always more accurate than E-BLUP, and it was also more accurate than either whole-genome Bayes A or whole-genome RKHS in 7 out of 12 comparisons. However, the bootstrap confidence intervals overlapped to some extent. Analyses performed with SNPs that were pre-selected using only sires from the low and high classes tended to have better predictive abilities (${\bar{r}}_{S}$ > 0.33) than analyses that involved an additional middle class, except for the setting with α = 0.30 (${\bar{r}}_{S}$ = 0.19). Analyses that included SNPs that were pre-selected based on information gain from three classes (low, medium and high) were more variable, although confidence bands overlapped. Four out of six analyses produced poorer predictions than either Bayes A or RKHS using all 3481 SNPs. This is probably due to the lower information gain obtained when separating sires into 3 classes. However, SNPs that were pre-selected using 3 classes and α = 0.25 had the best predictive ability, with an almost 4-fold improvement in prediction accuracy relative to E-BLUP. Pre-selection of SNPs reduces noise when measuring genomic differences, because non-informative SNPs are not considered. Furthermore, with pre-selected SNPs, this kernel placed more weight on informative SNPs. Other methods of pre-selecting SNPs are available and should be tested as well. Among these, the least absolute shrinkage and selection operator (LASSO; [33]), or its Bayesian counterpart [34] are appealing and yield predicted genomic values directly.

Bayes A and RKHS regression using all SNPs had similar predictive ability, even though these methods are very different from each other. Bayesian regression shrinks weakly informative SNPs towards zero, whereas RKHS regression makes weaker a priori assumptions and focuses on prediction of outcomes. Bayes A is also highly dependent on the prior distribution assigned to the variances of regression coefficients. Different scale parameters and degrees of belief for the *χ*^-2^(*υ*, *S*) distribution produced very different predictive abilities (only the best choice was shown in this study). The large *p*, small *n*, problem plays an important role in Bayes A, and posterior estimates are greatly affected by the choice of hyper-parameters in the prior distribution. Meuwissen *et al*. [2] chose their prior distribution for the variances of regression coefficients based on their simulation. This choice is not straightforward with real data. Hence, an extra layer is missing in the hierarchy of Bayes A. For example, markers on the same chromosome could be assigned the same prior variance, such that for chromosome *j*, the conditional posterior distribution of $\sigma_{j}^{2}$ would have *p*_*j*_+*r *degrees of freedom. The RKHS regression approach, on the other hand, is less dependent on priors, and it can also be implemented in a non-Bayesian manner. Nonetheless, two issues must be considered carefully in RKHS: the choice of the bandwidth parameter (*h*) and of the kernel (i.e., how to measure genomic differences). Generalized cross-validation or jackknife methods are broadly accepted for tuning smoothing parameters. In this study, the same bandwidth parameter was used in the training and testing sets, although tuning a specific parameter for each set is an option to be explored. However, measuring genomic differences (non-Euclidean distances) may be done in different ways, each yielding different predictive ability. The kernel described in this research performed best among several kernels that were tested. To be effective in prediction of phenotypes in future generations, a proper kernel function must be used. Furthermore, correct tuning of the smoothing parameter is needed. As knowledge on the genome increases, more suitable kernels may be designed. Since strong assumptions are used in parametric models (e.g., regarding dominance or epistasis), RKHS regression is expected to produce better predictions for complex traits because it may deal with crude, noisy, redundant and inconsistent information.

Computing times for the first 10,000 samples were 1398.6 and 110.88 CPU seconds for whole-genome Bayes A and whole-genome RKHS regression, respectively. Thus, the semi-parametric regression was 12.6 times faster, and computing time does not depend on the number of SNPs but, rather, on the number of animals that were genotyped. This is because the matrix of kernels is *n *× *n*, where *n *is the number of animals genotyped, irrespective of the number of SNPs. Computing time in Bayes A depends on the number of SNPs (or haplotypes) that are genotyped, because these influence the number of conditional distributions that must be sampled. Gibbs sampling may mix poorly, and methods including a Metropolis-Hastings step, such as Bayes B [2], might be prohibitive from a computational point of view when *p *is large. Further, convergence should be tested carefully in field data with Bayesian regression methods, as pointed out by ter Braak *et al*. [35].

Other authors have evaluated predictive ability of models including genomic information in different scenarios. Gonzalez-Recio *et al*. [8] analyzed a lowly heritable trait (chicken mortality) in a similar population using different parametric and non-parametric approaches. These authors found a higher predictive ability for RKHS regression than for other methods, including the Bayesian regression model proposed by Xu [5], which is similar to Bayes A. However, this study differed in some respects from the present research. For example, genomic differences between genotypes in the kernel utilized by Gonzalez-Recio *et al*. [8] were computed from only 24 pre-selected SNPs based on the filter-wrapper feature subset algorithm of Long *et al*. [9]. Also, predictive ability was assessed on current phenotypes, and not on phenotypes of future generations, as it was the case in the present study. Other authors using real data, such as a study in mice [36], found that methods incorporating genomic information produced more accurate phenotypic predictions than BLUP in a model with independent families.

## Conclusion

This research indicated that prediction accuracy of genetic evaluations can be enhanced by incorporating genomic data into breeding programs for a moderate heritability trait, such as FCR in broilers. This is one of the most important traits in the broiler industry from an economical point of view, and genome-assisted selection may help increase profitability in breeding and commercial flocks [37]. Reproducing kernel Hilbert spaces regression can handle a large number of markers without making strong assumptions, and the tandem approach of pre-selection of SNPs for subsequent use in RKHS regression seems to be an appealing approach, as found in this study. Pre-selection may be useful in the development of assays with fewer number of SNPs. Pre-selection of SNPs may be performed from a large battery of SNPs, genotyped on a restricted number of sires with a large number of progeny. Genotyping of animals on a greater scale may become more affordable if a smaller number of informative SNPs is included on a chip. Subsequently, semi-parametric methods can be used in conjunction with these SNPs to predict future phenotypes with high accuracy.

## Competing interests

OGR, DG, GJMR and KAW declare that they have no competing interests. AK is employed by Aviagen Ltd., which provided partial funding to the study.

## Authors' contributions

OGR participated in the design of the study and methods, the statistical analyses, discussions and drafted the manuscript. DG, GJMR and KAW participated in the design of the study, the statistical methods, discussions and helped revise the manuscript. AK gained access to the dataset, participated in preparing and editing data, discussions and helped revise the manuscript. All authors read and approved the final manuscript.
